# Socio-Economic and Demographic Correlates of Non-communicable Disease Risk Factors Among Adults in Saudi Arabia

**DOI:** 10.3389/fmed.2021.605912

**Published:** 2021-04-06

**Authors:** Mohammed Khaled Al-Hanawi, Mpho Keetile

**Affiliations:** ^1^Department of Health Services and Hospital Administration, Faculty of Economics and Administration, King Abdulaziz University, Jeddah, Saudi Arabia; ^2^Department of Population Studies, University of Botswana, Gaborone, Botswana

**Keywords:** adults, correlates, NCD risk factors, Saudi Arabia, socioeconomic

## Abstract

**Background:** Over the past two decades, Saudi Arabia has made significant improvements in its population's health standards. These improvements have been coupled with an increase in risk factors related to non-communicable diseases (NCD) and a dramatic shift in the burden of disease profile. This study aims to provide empirical evidence on the socio-economic and demographic correlates of NCD risk factors among adults in Saudi Arabia.

**Methods:** The data used for this study is secondary data derived from the Saudi Health Interview Survey (SHIS) conducted in 2013. The SHIS used a cross-sectional survey design to derive a multistage representative sample of adults to estimate the prevalence of NCD risk factors. Risk factors considered for analyses in this study were; current tobacco use, low fruit and vegetable consumption, low physical activity, overweight/obesity and hypertension. The survey covered all regions in Saudi Arabia using probability proportional to size measures. A total of 10,735 adults aged 15 years and above completed the survey questionnaire. Logistic regression analysis was conducted to examine the socio-economic and demographic correlates of NCD risk factors among adults in Saudi Arabia.

**Results:** The prevalence of NCD risk factors were as follows: current tobacco use, 12.1%; low fruit and vegetable consumption, 87%; low physical activity, 94.9%; overweight/obesity 65.1%; and hypertension, 37.5%. The multivariate analysis results indicate that significant correlates of overweight/obesity and hypertension were being female, a government employee, income level, and education levels. On the other hand, current tobacco use and low fruit and vegetable consumption were generally associated with age, self-employment and being a student. For lifestyle factors, overweight/obesity was high among individuals who reported low fruit and vegetable consumption, while hypertension was high among current tobacco users and overweight/obese adults. All comparisons were statistically significant at *p* < 0.05.

**Conclusions:** This study's findings indicate a high prevalence of chronic NCD risk factors in Saudi Arabia's adult population. This study implied that there is a need for a reduction in life-damaging behaviors among the adults through the adoption of healthy lifestyles such as physical activity and nutritious diets. Moreover, a reduction in the prevalence of chronic NCD risk factors among different socio-economic groups in Saudi Arabia through healthy lifestyles will have far-reaching results.

## Introduction

The evidence available across the world has shown that the majority of countries are experiencing an increase in NCD risk factors, which are observed to be prevalent in people of all age groups, among poor and non-poor people and across the gender divide (1). NCDs have been stated as being the leading cause of death worldwide, accounting for over 38 million (68%) of the world's 56 million deaths. More than 40% (16 million) of these deaths were for people under 70 years of age of and the majority of all NCD deaths, and of premature deaths, occur in low and middle-income countries (2). Previous research has indicated that the majority of the NCDs have common risk factors, which are often classified as being behavioral or biological (3). Behavioral risk factors include excessive alcohol consumption, tobacco use, physical inactivity and an unhealthy diet (4). These lifestyle factors lead to conditions such as metabolic or physiological changes, including overweight or obesity and raised blood pressure.

Among countries in Western Asia, the Kingdom of Saudi Arabia (KSA) has made significant progress in improving its population's health standards (5). These health improvements have led to a remarkable shift in the burden of disease profile, with a consequent transition away from communicable, maternal and perinatal factors toward NCDs. The causes of death due to diabetes and kidney diseases, cardiovascular diseases, maternal and neonatal disorders, respiratory infections and tuberculosis and nutritional deficiencies decreased between 2010 and 2017 in the KSA (6). Conversely, risk factors such as low physical activity, hypertension and high LDL cholesterol were observed to have continuously increased across the period (6). The increase in these metabolic and behavioral risk factors presents a major challenge in relation to the prevention and management of NCDs now and in the future.

There is little evidence on the socio-economic correlates of NCD risk factors in the KSA and it is important to understand the socio-economic differences in NCD risk factors. It has been remarked that NCD risk factors are extremely varied in their distribution across different socio-economic groups and their prevalence patterns rapidly change as societies develop (7–9). While some literature exists on the behavioral determinants of NCDs in the KSA (10, 11), little is known about the corresponding socio-economic correlates of NCD risk factors. As a result, greater insight into the risk factor correlates is important in relation to forming policies and to provide an understanding of the factors that are expected to contribute to the Kingdom's NCD risk factors inequalities. Therefore, this study aims to assess the socio-economic and demographic correlates of NCD risk factors in the KSA and, thereby, contribute to an increased understanding of the extent of the NCD risk factor burden and the socio-economic correlates.

## Materials and Methods

### Study Design and Sample

The data used for this study was secondary data derived from the Saudi Health Interview Survey (SHIS), which was conducted in 2013. The Saudi Ministry of Health (MOH) collaborated with the Institute for Health Metrics and Evaluation (IHME) and the University of Washington to conduct a nationally representative survey, which was used to estimate the prevalence of some of the NCD risk factors through an interview, and physical examination of study participants including weight, height and blood pressure which were measured by a trained professional (12).

A multistage stratified probability sampling design was employed to recruit the survey respondents to ensure that the survey findings were representative of the KSA population. The survey was conducted covering all 13 administrative regions in the KSA. A total of 12,000 households were selected and contacted and a total of 10,735 respondents aged over 15 were successfully interviewed, with a survey response rate of about 90%. A detailed description of the sampling methodology and data collection is available elsewhere (13, 14).

### Measurement of Variables

#### Outcome Variables

The outcome variables for this study are the NCD risk factors, namely current tobacco use, low fruit and vegetable consumption, low physical activity, overweight/obesity and hypertension. Tobacco use was measured as the percentage of respondents who currently use any tobacco products, such as cigarettes, cigars or pipes (15). This was a variable derived from the question which asked respondents whether they have ever used tobacco products. Those who responded with a “yes” were further asked whether they are currently using tobacco products. The coding of current tobacco use as a risk factor for NCDs was done based on the WHO STEPS Manual (9). The final variable was coded such that individuals who indicated that they were currently using tobacco products were given a code of 1 and 0 if otherwise. A total of 10,706 cases were used in the regression analysis, while 29 missing cases were excluded.

Consumption of fruits and vegetables was assessed in terms of the “number of servings” and this variable was computed such that the percentage of respondents who had less than five servings of fruit and/or vegetables on average per day were assessed as having low fruit and vegetable consumption. Those who had more than five servings of fruit/vegetables per day were considered to have good consumption consistent with WHO guidelines (16, 17). A total of 10,363 cases were used in the regression analyses for fruit and vegetable consumption, while missing cases were 372.

Respondents who, in a typical week, engaged in any physical activity, such as work, travel to and from places, and/or recreational activities, reported the number of days and the amount of time they spent doing those activities. Low physical activity evaluates the percentage of respondents who fail to meet the WHO physical activity for health recommendations. Adults aged between 15 and 64 should do at least 150 min of moderate to vigorous intensity physical activity during the week, or at least an hour of vigorous intensity physical activity or the equivalent (17, 18). Consequently, anyone who did not meet the cut-off as recommended by WHO was deemed to have low physical activity. Overall 10,187 cases were included in the logistic regression model.

Hypertension was computed by looking at the respondents' systolic and diastolic blood pressure levels. If the respondent's blood pressure was higher than 120/80 mm Hg on three separate occasions, they were categorized as being hypertensive and otherwise if the blood pressure was <120/80 mm Hg on any one occasion. The final variable was coded such that > 120/80 mm Hg on three occasions = 1 and <120/80 mm Hg on three occasions = 0. A total of 10,128 observations were analyzed in the logistic regression model, and 607 missing cases were excluded from the analysis.

The SHIS collected anthropometric information on height in meters (m) and weight in kilograms (kg) as per the WHO guidelines. Body Mass Index (BMI) was used to classify adults as underweight, normal weight, overweight and obese. BMI is calculated as an individual's weight in kilograms divided by the square of their height in meters (kg/m^2^) (19) and are categorized into underweight (BMI < 18.5), normal weight (18.5 ≤ BMI < 25), overweight (25 ≤ BMI < 30) and obese (BMI ≥ 30) (20). Overweight and obese was used to create a binary outcome variable that was coded as being overweight/obese (BMI ≥ 25) = 1 and not overweight/obese (BMI < 25) = 0. Overall a total of 10,421 respondents whose BMI was calculated as overweight/obese and not overweight/obese were included in the analysis, while 314 missing cases were excluded.

#### Explanatory Variables

The independent variables used for the analysis in this study were chosen based on the literature review and the availability of the socio-economic and demographic variables gathered by the 2013 SHIS. The following variables were collected during the survey: gender, age, marital status, education, work status, income level and region. Age was categorized as follows: 15–24, 25–34, 35–44, 45–54, 55–64 and 65+ years, while marital status was logged as currently married, or not married (divorced, never married, separated or widowed). Education was divided into below primary school, primary school, intermediate school, high school and higher education, while work status was categorized as government employee, homemaker, non-government employee, retired, self-employed, student and unemployed. For monthly income (Saudi Riyal, SR 1 = USD 0.27), the following categories were adopted: <3,000 SR, 3,000 to <5000 SR, 5,000 to <7,000 SR, 7,000 to <10,000, 10,000 to <15,000 SR, 15,000 to <20,000 SR, 20,000 to <30,000 SR and ≥30,000 SR.

### Data Analysis

Un-weighted data was used to study the participants' characteristics and, as part of the first step in the data analysis, a multicollinearity test of the outcome variables and explanatory variables was conducted to determine the presence of collinearity between them. The second step involved the use of descriptive statistics to assess the distribution and prevalence of NCD risk factors in the population. All explanatory variables that were not statistically significant were left out in the multivariable analysis. Confidence intervals were used to determine the statistical differences in the proportions of the outcome variables' categories. Lastly, logistic regression analysis was used to assess the association between the socio-economic and demographic variables and the NCD risk factors. The logistic regression models' results were presented as adjusted odds ratios (AOR) together with their 95% confidence intervals. All comparisons were considered to be statistically significant at *p* ≤ 0.05 level. Since a multistage stratified sampling procedure was used in the SHIS, the use of standard statistical methods to analyse the data would produce unreliable estimates of the desired parameters. As such, during the data analysis, a complex sample module from SPSS was used in order to account for the multiple stages of sampling. All the statistical analyses were conducted in SPSS version 25.

### Ethical Statement

This study was based on the use of secondary data from the SHIS. The survey was commissioned, funded or managed by the Saudi Arabia MOH, in collaboration with the Institute for Health Metrics and Evaluation (IHME) and the University of Washington which were all in charge of the ethical procedures. The data collection, archiving and use were done in compliance with the World Medical Association Declaration of Helsinki. All ethical procedures complied with the protocols for the protection of human subjects. The study protocol was approved by the Saudi Ministry of Health and its Institutional Review Board (IRB). The participants consented and agreed to participate in the study. There were two verbal consents which were administered at household and individual level. Only the respondents who agreed to the consent were recruited in the study. Participants were informed that taking part in the study is voluntary and they could withdraw at any time without given reason. Furthermore, they were also informed that the data would be used for the purpose of research at a future time point. All the personal identifiers were removed from that data, to allow for secondary data use. The MOH and the IHME granted the permission to use that data and thus no further clearance is necessary as this was done at the data collection phase.

## Results

### The Sample's Socio-Demographic Characteristics

Table 1 shows the sampled population's socio-economic and demographic characteristics. There were a slightly higher proportion of females (51.1%) than males (48.9%). The proportion of sampled respondents decreased with age and was highest in ages 25–34 years (25.7%). Moreover, a high proportion of respondents were currently married (65%), had a high school education (28%), were government employees (29.7%) and earned a monthly income of 3,000 to less 5,000 SR (20.1%).

**Table 1:** The sample's socio-economic and demographic characteristics.

**Variable**	***N***	**%**
**Gender**		
Male	5,249	48.9
Female	5,486	51.1
**Age**		
15–24	2,383	22.2
25–34	2,759	25.7
35–44	2,341	21.8
45–54	1,524	14.2
55–64	859	8.0
≥65	869	8.1
**Marital status**		
Married	6,978	65.0
Not Married	3,757	35.0
**Education**		
Below primary school	2,267	21.1
Primary school	1,158	10.8
Intermediate school	1,761	16.4
High school	3,005	28.0
Higher education	2,544	23.7
**Work status**		
Government employee	3,188	29.7
Homemaker	2,587	24.1
Non-government employee	462	4.3
Retired	773	7.2
Self-employed	440	4.1
Students	1,825	17.0
Unemployed	1,460	13.6
**Monthly income**		
<3,000 SR	2,029	18.9
3,000 to <5,000 SR	2,158	20.1
5,000 to <7,000 SR	1,943	18.1
10,000 to <15,000 SR	1,922	17.9
15,000 to <20,000 SR	1,159	10.8
20,000 to <30,000 SR	794	7.4
≥30,000 SR	730	6.8
**Total**	**10,735**	**100.00**

### Prevalence of NCD Risk Factors

**Figure 1:**
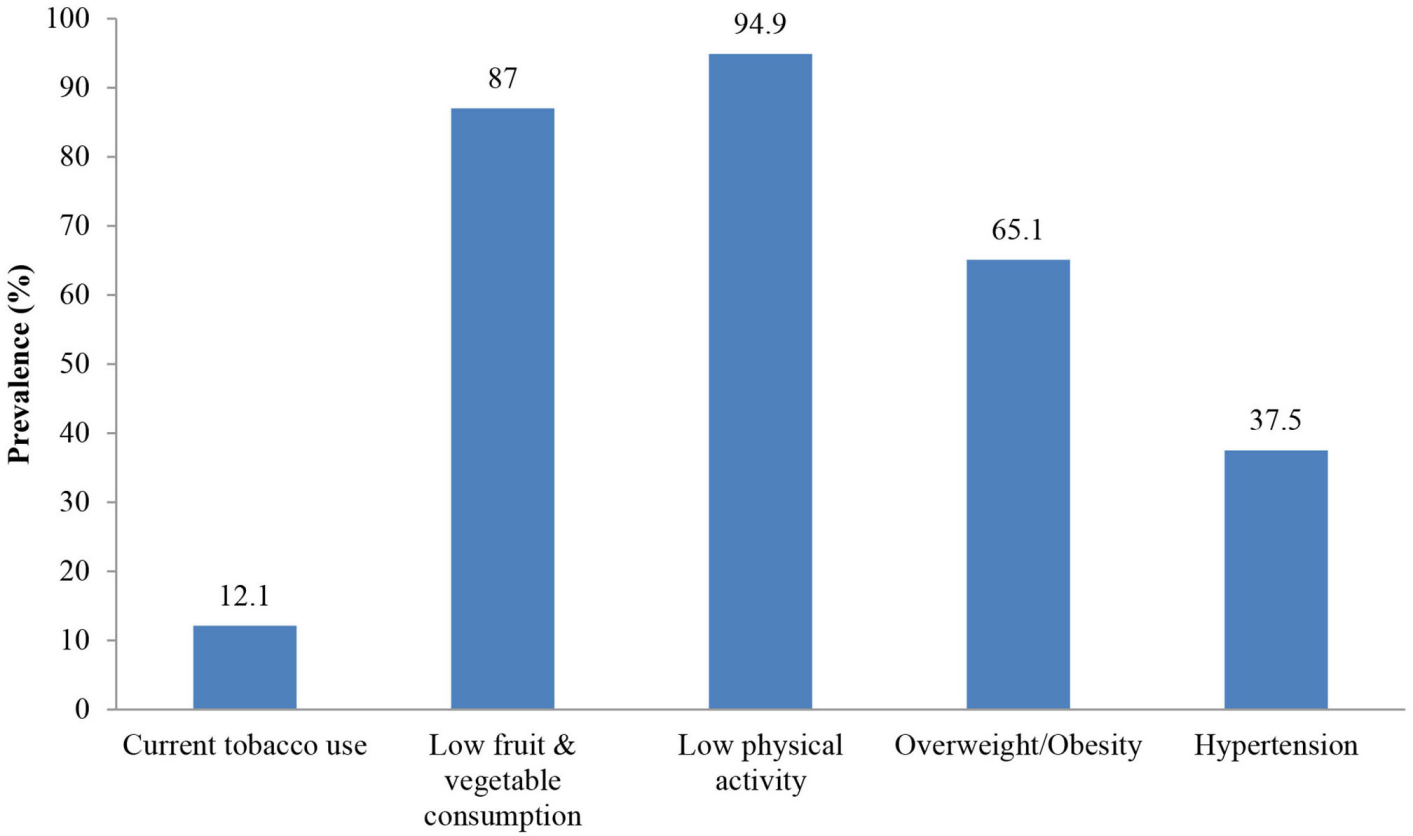
Prevalence of NCD risk factors in the sampled population (2013).

The most prevalent NCD risk factors in the sampled population were low physical activity (94.9%) and low fruit and vegetable consumption (87%) as it can be seen in Figure 1. More than two-thirds (65.1%) of the respondents were overweight/obese and over one-third (37.5%) had hypertension. The overall prevalence of current tobacco use was estimated at 12.1% in the sampled population.

The prevalence of current tobacco use (23.7 vs. 1.5%) and low fruit and vegetable consumption (87.2 vs. 86.8%), was significantly higher among males than females (Table 2). On the contrary, low physical activity (96 vs. 93.7%), overweight/obesity (67.5 vs. 63.1%) and hypertension (43 vs. 30.3%) were significantly high among females than males. Current tobacco use, overweight/obesity and hypertension significantly increased with age but, on the other hand, there was little variation in the prevalence of low fruit and vegetable consumption and poor physical activity, with the prevalence of these two risk factors slightly decreasing with increasing age

**Table 2:** Prevalence of NCD risk factors by the socio-economic and demographic factors (2013).

**Variable**	**Current tobacco use (*n* = 10,706)**	**Low fruit and vegetable consumption (*n* = 10,363)**	**Low physical activity (*n* = 10,187)**	**Overweight/obesity (*n* = 10,421)**	**Hypertension (*n* = 10,128)**
**Gender**					
Male	23.7	87.2	93.7	63.1	30.3
Female	1.5	86.8	96.0	67.5	43.0
*P*-value	0.000	0.492	0.000	0.000	0.000
**Age**					
15-24	8.0	91.9	96.6	37.2	16.2
25-34	14.8	87.4	94.5	65.5	25.2
35-44	15.1	86.0	94.4	77.3	36.8
45-54	14.4	82.4	94.2	80.6	52.1
55-64	12.5	84.1	94.5	80.8	68.6
65+	5.3	86.2	92.0	70.7	70.9
*P*-value	0.000	0.000	0.000	0.000	0.000
**Marital Status**					
Married	13.4	85.0	94.5	74.9	40.8
Not married	41.7	91.2	96.0	40.8	20.5
*P*-value	0.004	0.000	0.034	0.000	0.000
**Education**					
Below primary school	13.2	90.3	94.2	74.0	59.9
Primary school	12.8	90.3	94.2	64.4	39.5
Intermediate school	12.4	89.9	94.3	55.2	29.9
High school	16.3	88.0	94.5	60.9	30.0
Higher education	13.4	72.4	91.7	76.5	38.0
*P*-value	0.000	0.000	0.000	0.000	0.000
**Work status**					
Government employee	20.5	84.6	94.5	73.7	38.2
Homemaker	1.5	87.5	95.4	73.9	36.7
Non-government employee	28.1	83.0	90.5	64.4	35.6
Retired	17.7	84.3	81.8	73.7	70.9
Self-employed	28.5	83.1	91.3	69.0	47.8
Student	7.2	92.2	97.3	35.5	16.0
Unemployed	15.6	90.4	91.3	69.6	59.4
*P*-value	0.000	0.000	0.000	0.000	0.000
**Monthly income**					
<3,000 SR	10.4	92.5	95.2	57.2	44.2
3,000 to <5,000 SR	12.3	89.3	93.4	62.7	39.1
5,000 to <7,000 SR	15.2	86.4	94.3	67.4	35.3
7,000 to <10,000 SR	16.5	86.0	94.2	66.9	31.7
10,000 to <15,000 SR	15.2	81.8	95.5	71.2	36.4
15,000 to <20,000 SR	14.8	78.9	95.6	69.5	38.1
20,000 to <30,000 SR	18.0	72.2	96.4	69.9	35.9
≥30,000 SR	8.8	90.9	94.2	68.0	34.5
*P*-value	0.000	0.000	0.000	0.000	0.000

The prevalence of all NCD risk factors was significantly associated with marital status. For instance, overweight/obesity (74.9%) and hypertension (40.8%) were more prevalent among the married, while current tobacco use (41.7%), low fruit and vegetable consumption (91.2%) and low physical activity (96%) were more prevalent among non-married individuals. Current tobacco use was also significantly high among individuals with high school education (16.3%) than other education groups. Meanwhile the prevalence of low fruit and vegetable consumption, and low physical activity significantly decreased with education level and were highest among individuals with below primary education, while overweight/obesity was significantly high among individuals with higher education (76.5%) than other education categories.

For work status, current tobacco use was more prevalent among self-employed and non-government employees (28.5 vs. 28.1%, respectively), low fruit and vegetable consumption (92.2%) and low physical activity (97.3%) were more prevalent among students, overweight/obesity was more prevalent among government employees (73.7%) and homemakers (73.9%), while hypertension was more prevalent among retired individuals (70.9%). The proportion of individuals who reported current tobacco use was observed to be higher among individuals with an estimated monthly income of 20,000 to <30,000 SR (18%). Low fruit and vegetable intake (92.5%), low physical activity (95.2%) and hypertension (44.2%) were significantly high among individuals earning <3,000 SR. On the other hand, overweight/obesity prevalence was significantly high among individuals earning 10,000 to <15,000 SR (71.2%).

### Correlates of NCD Risk Factors Among Adults

Gender differences were observed for current tobacco use, with women less likely (AOR = 0.70, CI = 0.53–0.90) to report current tobacco use compared to their male counterparts. There was no variation in the respondents' age, marital status, education level, monthly income and current tobacco use in the logistic regression model (Table 3). Non-government employees (AOR = 0.61, CI = 0.40–0.93) and students (AOR = 0.39, CI = 0.27–0.53) were less likely to report current tobacco use, while self-employed individuals (AOR = 2.41, CI = 1.15–5.08) were more than 2 times more likely to report current tobacco use than unemployed individuals.

**Table 3:** Adjusted odd ratios for the association between the socio-economic, behavioral factors and NCD risk factors (2013).

**Variable**	**Current tobacco use**		**Low fruit and vegetable consumption**		**Low physical activity**		Overweight/obesity*		Hypertension*	
	**AOR**	**95% CI**	**AOR**	**95% CI**	**AOR**	**95% CI**	**AOR**	**95% CI**	**AOR**	**95% CI**
**Socio-economic factors**										
**Gender**										
Male	1.00		1.00		1.00		1.00		1.00	
Female	0.70***	(0.53–0.90)	0.86	(0.73–1.02)	1.61***	(1.15–2.25)	1.20***	(1.07–1.35)	1.54***	(1.13–2.14)
**Age**										
15–24	1.00		1.00		1.00		1.00		1.00	
25–34	1.12	(0.84–1.52)	0.74***	(0.61–0.91)	1.05	(0.64–1.72)	1.86***	(1.55–3.42)	1.87***	(1.61–2.18)
35–44	1.07	(0.78–1.50)	0.64***	(0.52–0.79)	1.05	(0.61–1.78)	5.53***	(4.81–6.35)	3.26***	(2.81–3.79)
45–54	1.03	(0.73–1.46)	0.39***	(0.31–0.49)	1.10	(0.62–1.95)	6.87***	(5.79–8.14)	5.93***	(5.01–7.00)
55–64	0.83	(0.56–1.26)	0.35***	(0.26–0.45)	1.43	(0.68–3.00)	7.69***	(6.18–9.57)	10.6***	(8.68–13.1)
65+	0.27	(0.17–18.4)	0.29***	(0.22–0.40)	0.95	(0.41–2.20)	4.53***	(3.65–5.63)	10.2***	(8.21–12.7)
**Marital status**										
Not married	4.45	(0.51–39.5)	0.42***	(0.23–0.77)	0.85	(0.38–1.88)	0.51***	(0.38–0.69)	0.52***	(0.35–0.77)
Married	1.00		1.00		1.00		1.00		1.00	
**Education**										
Below primary school	1.01	(0.56–1.84)	3.75***	(2.02–6.94)	2.35	(0.92–5.99)	1.07	(0.63–1.80)	1.07	(0.63–1.81)
Primary school	0.63	(0.34–1.19)	2.02***	(1.12–3.64)	1.91	(0.76–4.80)	0.71	(0.42–1.19)	1.35	(0.68–1.89)
Intermediate school	0.75	(0.45–1.30)	2.51***	(1.37–4.60)	1.92	(0.89–4.17)	0.75	(0.46–1.22)	1.04	(0.63–1.72)
High school	0.73	(0.06–7.30)	1.68	(0.94–2.98)	1.55	(0.69–3.44)	0.31	(0.05–1.68)	1.08	(0.65–1.79)
Higher education	1.00		1.00		1.00		1.00		1.00	
**Work status**										
Government employee	0.18	(0.01–1.82)	1.02	(0.22–4.70)	1.23	(0.52–2.38)	1.22***	(1.02–1.46)	1.28***	(1.06–1.54)
Homemaker	0.99	(0.75–1.81)	1.32	(1.03–1.69)	0.34***	(0.19–0.60)	1.37	(0.46–4.11)	2.28	(0.75–6.90)
Non-government employee	0.61***	(0.40–0.93)	0.94	(0.75–1.19)	0.34***	(0.18–0.63)	1.16	(0.98–1.36)	1.23***	(1.03–1.46)
Retired	1.30	(0.95–1.80)	0.85	(0.61–1.20)	0.25***	(0.13–0.48)	1.03	(0.80–1.33)	1.12	(0.86–1.47)
Self-employed	2.41***	(1.15–5.08)	1.27	(0.20–5.69)	0.11***	(0.03–0.43)	0.65	(0.25–1.70)	1.61***	(1.25–2.06)
Student	0.39***	(0.27–0.53)	1.41***	(1.01–1.97)	0.26***	(0.13–0.50)	0.97	(0.76–1.25)	1.28	(0.98–1.66)
Unemployed	1.00		1.00		1.00		1.00		1.00	
**Monthly income**										
<3,000 SR	1.00		1.00		1.00		1.00		1.00	
3,000 to <5,000 SR	0.94	(0.73–1.25)	0.41	(0.31–0.55)	1.58	(0.65–1.78)	1.33	(0.95–1.87)	0.88	(0.72–1.07)
5,000 to <7,000 SR	0.92	(0.68–1.31)	0.37	(0.27–0.51)	1.01	(0.65–1.73)	1.28***	(1.03–2.03)	0.95	(0.75–1.21)
7,000 to <10,000 SR	1.15	(0.74–1.69)	0.29	(0.20–0.44)	0.91	(0.51–1.77)	1.49***	(1.18–1.89)	0.79	(0.56–1.12)
10,000 to <15,000 SR	0.81	(0.64–1.06)	0.66	(0.50–1.87)	0.89	(0.49–1.72)	1.49***	(1.25–1.77)	0.94	(0.79–1.20)
15,000 to <20,000 SR	0.43	(0.25–2.75)	0.93	(0.54–1.58)	0.98	(0.57–1.79)	1.53***	(1.28–1.82)	0.66***	(0.47–0.94)
20,000 to <30,000 SR	0.62	(0.43–0.93)	0.53	(0.40–0.69)	0.81	(0.23–1.91)	1.63***	(1.25–2.13)	0.88	(0.73–1.06)
≥30,000 SR	0.61	(0.45–0.85)	0.95	(0.71–1.28)	2.43***	(1.35–4.36)	1.33***	(1.01–2.08)	0.85	(0.64–1.11)
**Behavioral factors**										
**Current tobacco use**										
Yes							0.62***	(0.45–0.79)	1.36***	(1.07–1.69)
No							1.00		1.00	
**Fruit and vegetable intake**										
Low							1.30***	(1.15–1.49)	0.88	(0.78–1.00)
High							1.00		1.00	
**Physical activity**										
Low							0.99	(0.79–1.24)	0.89	(0.71–1.11)
High							1.00		1.00	
**Overweight/obese**										
Yes									2.21***	(2.01–2.43)
No									1.00	

*** *Statistically significant at 95% confidence level (CI)*.

* *Overweight/obesity and hypertension were associated with other NCD risk factors. As a result their analysis was extended to assess such association*.

For low fruit and vegetable consumption, the odds were significantly low among other age groups than among individuals aged 15–24 years and among non-married individuals (AOR = 0.42, CI = 0.23–0.77), than those who were married. The odds of low fruit and vegetable consumption were significantly high among individuals with below primary (AOR = 3.75, CI = 2.02–6.94), primary (AOR = 2.02, CI = 1.12–3.64) and intermediate school (AOR = 2.51, CI = 1.37–4.60) education level than among those with a higher education level. Similarly, the odds of low fruit and vegetable consumption were observed to be high among students compared with unemployed individuals.

The odds of low physical activity were significantly high among females (AOR = 1.61, CI = 1.15–2.25) than males and were also highest among individuals with a monthly income ≥30,000 SR (AOR = 2.43, CI = 1.35–4.36). Meanwhile the odds of low physical activity were significantly low among home-makers (AOR = 0.34, CI = 0.19–0.60), non-government employees (AOR = 0.34, CI = 0.18–0.63), retired (AOR = 0.25, CI = 0.13–0.48), self-employed (AOR = 0.25, CI = 0.13–0.48) and students (AOR = 0.26, CI = 0.13–0.50) than among unemployed individuals.

The results indicated that females were 1.2 times (AOR = 1.20, CI = 1.07–1.35) more likely to be overweight/obese than males. The odds of being overweight/obese increased with age until the age of 55–64 years (e.g., 25–34 years, AOR = 1.86, CI = 1.55–3.42, 35–44 years, AOR = 5.53, CI = 4.81–6.35, 45–54 years, AOR = 6.87, CI = 5.79–8.14) and declined in those aged over 65 years (AOR = 4.53, CI = 3.65–5.63). Non-married individuals were less likely (AOR = 0.51, CI = 0.38–0.69) to be overweight/obese compared to their married counterparts. Meanwhile, the odds of being overweight/obese were significantly high among government employees than the unemployed, individuals with a monthly income of 5,000 to <7,000 SR (AOR = 1.28, CI = 1.03–2.03), 7,000 to <10,000 SR (AOR = 1.49, CI = 1.18–1.89), 10,000 to <15,000 SR (AOR = 1.49, CI = 1.25–1.77), 15,000 to <20,000 SR (AOR = 1.53, CI = 1.28–1.82), 20,000 to <30,000 SR (AOR = 1.63, CI = 1.25–2.13) and ≥30,000 SR (AOR = 1.33, CI = 1.01–2.08) than among individuals earning a monthly income of <3,000 SR. Furthermore, an association was found between being overweight and behavioral factors. For instance, people who reported low fruit and vegetable intake were more likely (AOR = 1.36, CI = 1.07–1.69) to be overweight/obese, compared to those with high vegetable intake, while current tobacco users were less likely (AOR = 0.62, CI = 0.45–0.79) to be overweight/obese compared to non-tobacco users.

Females were more likely (AOR = 1.54, CI = 1.13–2.14) to be hypertensive compared to males and the odds of hypertension prevalence significantly increased with age. For example, individuals aged 25–34 (AOR = 1.87, CI = 1.61–2.18), 35–44 (AOR = 3.26, CI = 2.81–3.79), 45–54 (AOR = 5.93, CI = 5.01–7.00), 55–64 (AOR = 10.6, CI = 8.68–13.1) and ≥ 65 years (AOR = 10.2, CI = 8.21–12.7) were more likely to have hypertension compared to those aged 15–24 years. It was also observed that government employees (AOR = 1.28, CI = 1.06–1.54), non-government employees (AOR = 1.23, CI = 1.03–1.46) and self-employed (AOR = 1.61, CI = 1.25–2.06) individuals were more likely to have hypertension. For behavioral factors, it was observed that current tobacco users (AOR = 1.36, CI = 1.07–1.69) and overweight/obese (AOR = 2.21, CI = 2.01–2.43) individuals were more likely to have hypertension compared to non-tobacco users and non-overweight/obese people, respectively. Quite conversely, there was no statistically significant association between hypertension, low fruit and vegetable consumption and low physical activity.

## Discussion

The KSA has experienced marked demographic, nutritional and epidemiological transitions in the past three decades. As a result, the prevalence of NCD risk factors has disproportionately increased and led to NCDs becoming the main contributor to the burden of disease. Overall the study findings indicate that modifiable NCD risk factors—low physical activity, low fruit vegetable and consumption, current tobacco use, overweight/obesity and hypertension—are significantly high in the adult population of the KSA, which is accounted for by the significant economic and political progress that contributes vastly to different changes in disease risk factors and burdens. Diseases related to over-nutrition and sedentary lifestyles commonly found in the high-income countries are now more prevalent in the KSA and the Western Asia region in general (21, 22).

Adjusted analyses indicate that there was no variation in tobacco use for different socio-economic factors except for gender, employment status. For instance, we found that tobacco use was significantly prevalent among men and self-employed adults and this corroborates findings from studies in Italy (23), Germany (24), Brazil (25), South Africa (26) and Zambia (27), which also found that the tobacco smoking odds were highest among men, compared to women. In Saudi Arabia like in most countries there is a social stigma against smoking by women, which is seen as shameful (28). Similar findings were observed by other study in other Arab countries (29). This is different from Western societies where female smoking is more common (30).

There was no variation in low fruit and vegetable consumption between men and women. This finding is quite revealing and is not consistent with some other studies. For instance, Rasmussen, Krølner (31) reviewed fruit and vegetable intake in several countries and found that most studies (27 out of 49) showed gender differences in which men had a lower intake of fruits and/or vegetables than women. We also found that low fruit and vegetable consumption was significantly low among individuals of higher ages and those who were not married. On the other hand, low fruit and vegetable consumption was positively associated with low education level and being a student. This finding corroborates previous studies, which have shown that education has an effect on income/socio-economic status and, as a result, tends to have one of the strongest influences on fruit and vegetable intake (32–35).

The findings indicate that women were more likely to report poor physical activity compared to men, which is an expected finding because studies in both high-income and low-income settings have generally shown that men are physically active compared to women (36, 37). This finding is, in part, explained by the fact that women often lack time due to demands such as child-rearing, household duties, lack of motivation and health problems (38). Other reasons may include gender stereotypes that women should act as “queens” and should only do domestic chores. Other studies have also suggested that women were more likely to be physically inactive in Muslim countries and seemed to imply that religion constitutes an obstacle to physical activity (39).

Consistent with findings from most studies in low and middle-income countries, we found that the odds of being overweight/obese were significantly high among women. Overweight/obesity among women has been linked with biological, physiological and lifestyle factors. According to Templeton (40), being overweight or obese among women can be linked with changes in the reproductive cycle, with reduced fertility, as well as with a heightened risk of polycystic ovarian syndrome (PCOS) and infrequent or no ovulation. One of the plausible explanations of why overweight/obesity was significantly high among women in this study is that the sampled women were physically inactive. We also found that overweight/obesity increased with age and income. This finding corroborates data from large population studies that demonstrate that mean body weight and BMI gradually increase during most of people's adult lives and reach peak values at 50–59 years old in both sexes. After 60 years of age, mean body weight and BMI have a tendency to decrease (41).

For behavioral factors, overweight/obesity was positively associated with low fruit and vegetable intake. The available limited evidence corroborates this finding and suggests that, when consumed as part of a healthy diet, fruits and vegetables might help to prevent weight gain and reduce the risk of overweight or obesity (42). Consistent with other studies, we found that adults who currently use tobacco were less likely to be overweight or obese, which is because the nicotine found in tobacco products is stated to suppress appetite. However, unlike other studies' findings, it was observed that low physical activity was not a significant correlate of overweight/obesity.

Women were more likely to have been diagnosed with hypertension compared to men. Similarly, other studies have also found that the burden of hypertension showed gender variations, with women more likely to report hypertension than men (43–45). Furthermore, it is plausible that lower levels of hypertension among men than women in KSA may also be associated with the poor physical activity associated with changing lifestyles among women. It was also found that the odds of being diagnosed with hypertension increased with age. For example, respondents aged 55 years and above were 10 times more likely to have hypertension than the young adolescents. This is consistent with previous studies, which also found that hypertension increases with age in both developed and developing countries (46).

Hypertension was also observed to be high among government employees, non-government employees and self-employed individuals. The odds of hypertension were found to be high among current tobacco users and overweight/obese individuals. There is no consensus regarding the role of smoking on hypertension in generally healthy people and the effect of smoking cessation on hypertension remains unclear, with contradictory findings (47–49). A meta-analysis of 62 randomized controlled trials conducted by Aubin, Farley (50) found that smoking cessation was associated with a mean weight gain after 1 year of abstinence.

This study's main strength is that it uses a relatively large sample that is nationally representative. Moreover, the study utilizes a standard chronic disease risk surveillance methodology (WHO STEPs) and the findings are seen to be comparable to those from other settings and countries. This study's main limitation is that a cross-sectional design was used and, therefore, it was not feasible to determine the causal relationship between explanatory variables and NCD risk factors. Furthermore, the data for the study was collected seven years ago. However, this study provides vital insights into the socio-economic correlates of NCD risk factors using national survey data. Additionally, as the SHIS does not cover non-Saudi residents, results of this study are limited to the Saudi nationals.

## Conclusion

This study shows a high prevalence of NCD risk factors in the KSA adult population. Overweight/obesity and hypertension were significantly associated with the respondents' age, marital status, work status and monthly income. Overweight/obesity was observed to be high among individuals, who reported low fruit and vegetable consumption, while hypertension was high among individuals who were current tobacco users and were overweight/obese. This study implies that there is a need to focus on the reduction of life-damaging behaviors among adults through the adoption of healthy lifestyles such as physical activity and healthy diets.

## Data Availability Statement

The datasets generated and/or analyzed during the current study are not publicly available due to privacy, confidentiality and other restrictions. Access to data can be gained through the Ministry of Health in Saudi Arabia. Requests to access these datasets should be directed to www.moh.gov.sa.

## Ethics Statement

This study was based on the use of secondary data from the SHIS. The survey was commissioned, funded or managed by the Saudi Arabia MOH, which was in charge of the ethical procedures. The data collection, archiving and use were done in compliance with the World Medical Association Declaration of Helsinki. All ethical procedures complied with the protocols for the protection of human subjects. The study protocol was approved by the Saudi Ministry of Health and its Institutional Review Board (IRB). The participants consented and agreed to participate in the study. There were two verbal consents which were administered at household and individual level. Only the respondents who agreed to the consent were recruited in the study. Participants were informed that taking part in the study is voluntary and they could withdraw at any time without given reason. Furthermore, they were also informed that the data would be used for the purpose of research at a future time point. All the personal identifiers were removed from that data, to allow for secondary data use. The MOH granted the permission to use that data and thus no further clearance is necessary as this was done at the data collection phase.

## Author Contributions

All authors made substantial contributions to conception and design, acquisition of data, or analysis and interpretation of data took part in drafting the article or revising it critically for important intellectual content gave final approval of the version to be published and agree to be accountable for all aspects of the work.

## Conflict of Interest

The authors declare that the research was conducted in the absence of any commercial or financial relationships that could be construed as a potential conflict of interest.
